# The Impact of Social Presence on Purchase Intentions of Knowledge Products Among Knowledge-Based Short Video Users: A Moderated Mediation Model

**DOI:** 10.3390/bs15081140

**Published:** 2025-08-21

**Authors:** Can Zheng, Shuai Ling, Yarong Huang, Xinxiang Li

**Affiliations:** 1 School of Media, Jiangsu Second Normal University, Nanjing 210013, China; canzheng1228@jssnu.edu.cn; 2 College of Art and Design, Nanjing Forestry University, Nanjing 210037, China; 3 Department of Industrial Design, Hongik University, Seoul 04066, Republic of Korea; huangyarong@g.hongik.ac.kr; 4 Department of Design and Manufacturing Engineering, Jeonbuk National University, Jeonju 54896, Republic of Korea; lixinxiang@jbnu.ac.kr

**Keywords:** knowledge-based short videos, social presence, cognitive engagement, expectations, knowledge anxiety, purchase intention

## Abstract

Despite the rapid growth of knowledge-based short videos, monetizing this content remains a significant challenge. Grounded in social presence theory, this study investigates how social presence influences users’ purchase intentions by incorporating the mediating effects of cognitive engagement and expectation, as well as the moderating effects of knowledge anxiety. Using data from 663 users of knowledge-based short videos in China, the proposed model demonstrates strong explanatory power for purchase intention (R^2^ = 54.6%). The findings show that social presence significantly enhances users’ intention to purchase knowledge products by fostering cognitive engagement and expectations, creating a serial mediation effect. Furthermore, knowledge anxiety positively moderates the impact of social presence on purchase intention, with a more pronounced effect for individuals with higher anxiety. This research provides a novel theoretical perspective for understanding user behavior in knowledge-based short videos and offers practical guidance for platforms and creators to enhance monetization.

## 1. Introduction

As the rapid growth of Internet video traffic reaches its peak, users focus gradually shifts from entertainment content to more knowledge-based and educational material. Knowledge-based short videos (KBSVs) are increasingly gaining traction, typically ranging from 15 s to several minutes. Unlike entertainment content that relies on sensory stimulation, KBSV emphasizes on dissemination of scientific knowledge, learning skills, and personal experiences (H. Chen et al., 2021). According to TikTok’s research report, “Short Video Platforms Co-create a New Ecosystem for Knowledge Dissemination”, the platform published over 337 million KBSVs in a single month as of January 2024—a 30% increase compared to July 2023. These KBSVs span over 20 fields, including science communication, health, history and culture (Short Video Platforms Co-create a New Ecosystem for Knowledge Dissemination, 2024). The report also notes that 95% of respondents acquire knowledge through short videos, and 55% of the knowledge they encounter weekly comes from such content (Short Video Platforms Co-create a New Ecosystem for Knowledge Dissemination, 2024). Further research from Tsinghua University indicates that over 70% of users actively engage by liking, saving, and interacting with KBSVs, fostering a positive feedback loop of “creation-sharing-participation” (Learning Unbound, Shared Horizons: Short Video & Live Streaming for Knowledge Learning, 2024). This cyclical process describes how users, inspired by KBSV, actively create original content and share it with others. This sharing stimulates further attention and interaction from other users, which in turn encourages broader participation through discussions or the creation of secondary content. In 2021, the number of knowledge-based creators on Bilibili rose by 92%, with over 183 million users watching related videos. Among the top 100 creators, 14 were from the knowledge sector, collectively amassing over 3 billion views (F. Li & Li, 2022). KBSVs make up 43.8% of all knowledge-based videos on YouTube (Violot et al., 2024). Kuaishou’s “Kuaipedia”, a multimodal encyclopedia linking short videos with knowledge points, illustrates the significant categorization and growth of KBSVs on the platform (Pan et al., 2022). This trend aligns with the rise in specialized online learning platforms like Coursera and Udemy, which increasingly utilize short videos for microlearning.

Meanwhile, related studies from the United States and Denmark suggest that short videos effectively meet users’ information needs and contribute to improved digital literacy, collaborative learning, and informal education (Bucknell Bossen & Kottasz, 2020; Boffone, 2022). These platforms allow users to participate in all stages of knowledge construction (Nguyen & Diederich, 2023). Clearly, short video platforms serve as powerful technological intermediaries, constructing a highly efficient knowledge dissemination network. Through KBSVs, they facilitate effective connections between creators, content and users.

While KBSVs are experiencing rapid development, they also encounter numerous challenges. First, KBSVs struggle with converting commercial value. The revenue of KBSV creators primarily relies on two factors: platform incentives and the sales of knowledge products (such as online courses, video lectures, e-books, paid Q&A, and Knowledge Software Membership) (S. Jiang et al., 2024). Apart from the temporary boost provided by platform incentives, the sale of knowledge products serves as the core income source for KBSV creators. As a unique type of experiential product, knowledge products often confront the issue of information asymmetry, which significantly hampers their sales (Fang et al., 2021). Additionally, empirical research on TikTok’s knowledge-sharing ecosystem indicates that an abundance of free knowledge resources can lead to structural contradictions. When content creators attempt to set reasonable prices for their expertise, they frequently encounter resistance from user habits and the prevailing market environment, complicating the monetization of knowledge (H. Shi et al., 2023). Second, KBSVs face challenges associated with content lifespan. Unlike traditional educational content, KBSVs typically have a limited shelf life and often lose relevance within a few weeks (Ye et al., 2021). Consequently, KBSVs not only grapple with converting commercial value but also contend with short revenue cycles. In light of these significant challenges, user purchase intention—an essential indicator of social media marketing effectiveness—requires prioritized attention (Torres et al., 2018). Understanding the mechanisms that influence users’ intentions to purchase knowledge products in KBSV can significantly contribute to establishing a distinct competitive advantage for both platforms and content creators.

The academic community has conducted extensive research on the topic of knowledge product purchase intentions. For example, Liu et al. found that social value perception, utilitarianism, and hedonism directly influence consumers’ ongoing purchase intentions for knowledge products, while content and service quality exert an indirect effect (Liu et al., 2023). This suggests that intrinsic value perception is a fundamental factor driving knowledge payment behavior. Additionally, Zhang et al.’s study, utilizing an intermediary model analysis of transaction data, demonstrates that information asymmetry indirectly hampers the payment rate for knowledge products by heightening perceived uncertainty and diminishing price acceptance (X. Zhang et al., 2023). This highlights the significant impact of the external information environment on consumers’ value judgments and decision-making processes. To gain a more comprehensive understanding of how consumers evaluate knowledge products, Su et al. proposed a model grounded in prospect theory, indicating that consumer experience value serves as the primary criterion for assessing knowledge payment products (Jiafu et al., 2024). Furthermore, Zhao et al. discovered that price levels positively influence the trust effect, and that users’ decisions to pay for knowledge products are favorably affected by the reputation, competency, and integrity of content creators (Y. Zhao et al., 2018). This underscores the importance of creator characteristics and platform pricing strategies in facilitating knowledge payment.

The existing literature has established a preliminary theoretical framework regarding the mechanisms underlying knowledge product purchase intentions. Most studies concentrate on two dimensions: consumer value perception and content creator characteristics. It is important to acknowledge that current research is limited in its adaptability to various scenarios and that there are significant gaps in the empirical exploration of KBSV as an emerging content form, with no differentiated theoretical explanatory framework developed thus far.

Social presence, a critical analytical theory for digital interaction contexts, has garnered considerable attention in social media and e-commerce sectors. A higher level of social presence typically correlates with stronger emotional connections and social interactions, which can influence users’ purchase intentions in social media, e-commerce, and virtual environments (N. Li et al., 2024). Consequently, given the unique “short, flat, and fast” communication characteristics and interactive attributes of KBSV, this study incorporates social presence theory into its research framework. Additionally, existing studies have neglected the significant influence and interaction of cognitive engagement and expectations. Research shows a strong link between cognitive engagement and consumer behavior (Cheung et al., 2021). This relationship is especially evident in learning scenarios, where cognitive engagement is considered a key factor affecting outcomes (C. Huang et al., 2023). This aligns with the theme of KBSV marketing and is, therefore, included in this study.

Expectations are consistent with consumer prediction paradigms that emphasize perceptions of specific product value aspects, thus warranting the inclusion of expectations in the social presence-purchase intention model framework of this study. Moreover, previous studies have overlooked the significant impact of knowledge anxiety on KBSVs. In an era characterized by rapid advancements in artificial intelligence, exponential information growth, and significantly shortened knowledge renewal cycles, this sense of anxiety has become increasingly pronounced, emerging as an important psychological factor influencing individuals’ information acquisition and learning behavior (Zhenlei et al., 2024a). Therefore, integrating knowledge anxiety into the KBSV research framework is theoretically and practically significant for a comprehensive understanding of user motivations and behavior patterns.

In KBSVs, cognitive engagement and expectations are critical factors influencing user behavior. Cognitive engagement pertains to the actual investment and depth of information processing by users while viewing KBSVs, which subsequently affects their perception of the “certainty” value of short video content. Conversely, expectations are typically grounded in users’ prior assessments of knowledge products and influence their evaluation of the “possibility” of potential value. Currently, there is a lack of research that systematically integrates these variables to analyze their impact on user purchase intentions. Therefore, this study aims to develop a model examining the influence of social presence on users’ purchase intentions for knowledge products in KBSVs, focusing on the dimensions of “certainty” and “possibility”.

**Figure 1:**
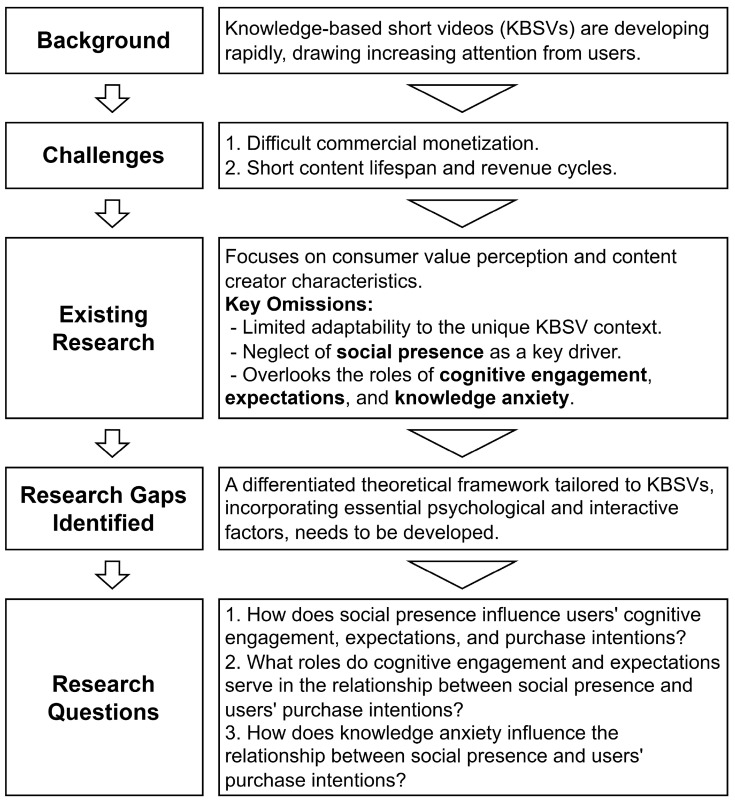
Diagram of the Introduction Structure.

Based on the preceding background and discussion, as shown in Figure 1, this study aims to investigate the following key research questions:

1. How does social presence influence users’ cognitive engagement, expectations, and purchase intentions?
2. What roles do cognitive engagement and expectations serve in the relationship between social presence and users’ purchase intentions?
3. How does knowledge anxiety influence the relationship between social presence and users’ purchase intentions?

This study seeks to elucidate the formation mechanism of users’ purchase intentions for knowledge products within the context of KBSV by developing a comprehensive model. It offers a novel perspective on user psychology research in this domain. The study posits that social presence serves as a critical antecedent variable that enhances users’ purchase intentions. Its operative mechanism involves fostering users’ cognitive engagement, which actively shapes their expectations regarding knowledge products and ultimately drives their purchase intentions. Furthermore, this research identifies the significant moderating effect of knowledge anxiety within the model, providing valuable insights for platforms and content creators on effectively leveraging social presence to improve user payment conversion rates. This work not only addresses existing gaps in the research concerning the underlying psychological motives influencing payments for KBSV knowledge products but also offers a framework and innovative strategies for short video platforms to optimize user experience and enhance competitive advantage.

The remainder of this paper will systematically organize relevant theories to formulate hypotheses and models, followed by a discussion of the results derived from the questionnaire data. Conclusions will then be drawn, and the limitations of this study, along with suggestions for future research, will be presented.

## 2. Theoretical Background

### 2.1. Social Presence

Social presence refers to how users perceive the presence and interaction of others when communicating through technology (Kreijns et al., 2021). It serves as a crucial indicator for assessing the “sense of real presence” in a social environment (Oh et al., 2018; Short et al., 1976). Since its introduction by Short et al., the concept has expanded from telecommunications to encompass online education, business, social media, and various other domains (Argo et al., 2005; Cui et al., 2012; Zheng et al., 2024). It is considered a crucial factor influencing judgments and decisions in media environments (Hawkins & Vandervelde, 2020). Research consistently confirms that social presence is essential for optimizing online experiences (Gefen & Straub, 2004). In the educational sector, a high level of social presence can significantly enhance learner interaction, satisfaction, and learning outcomes while mitigating the feelings of isolation associated with online learning (J. Kim et al., 2011; So & Brush, 2008; Y. Zhang et al., 2024). Additionally, learners’ perceptions of social presence are influenced by their characteristics (Mykota, 2015). In a business context, social presence can enhance customer engagement and brand loyalty, thereby influencing final behavioral decisions (Lim et al., 2015; Yang et al., 2021). For remote teams, social presence fosters trust and cooperation, ultimately improving team performance. As emphasized by social information processing theory, even non-face-to-face communication can cultivate strong interpersonal relationships over time (Walther, 1992). Emerging technologies, such as virtual reality, have opened new avenues for research on social presence (Sun et al., 2019; Ying et al., 2021). The immersive experience provided by virtual reality enhances users’ perceptions of co-presence and can facilitate the development of social support within virtual social environments (van Brakel et al., 2023).

Despite the prevalence of single-dimensional analyses in existing studies, social presence is fundamentally a multidimensional construct shaped by the integration of various factors (Z. Huang et al., 2022). The conceptualization of social presence dimensions varies across theoretical frameworks within the academic community. Biocca et al. identify co-presence, psychological involvement, and behavioral engagement as key dimensions of social presence (Biocca et al., 2003), highlighting perceptions of physical space, emotional cognitive processing, and behavioral feedback, respectively. According to Kathy et al., social presence encompasses three primary dimensions: consciousness, emotion, and cognition (Shen & Khalifa, 2008). Despite these theoretical variations, Kim et al. contend that in mediated environments, co-presence, and psychological involvement serve as the core dimensions with universal applicability (J. Kim et al., 2016). Accordingly, this study defines social presence through a two-dimensional framework that includes co-presence and psychological engagement. Co-presence emphasizes the physical dimension of spatial perception, while psychological involvement addresses the psychological aspect of social interaction. Collectively, they form the collaborative structure of social presence in KBSV scenarios. Co-presence refers to the perceived presence of others in a media environment, wherein individuals experience an embodied sense of togetherness within the same spatiotemporal context, thus creating an interactive sense of spatial proximity. Psychological involvement denotes the profound immersion experienced during social interactions, characterized by emotional connections (Animesh et al., 2011; Biocca et al., 2003).

### 2.2. Cognitive Engagement

Cognitive engagement is the degree of attention, concentration, and depth of thinking that individuals demonstrate during activities involving information processing, such as learning and decision-making (Dessart et al., 2016; Hollebeek et al., 2014). This concept, rooted in educational psychology, is recognized as a crucial element of the educational experience (Barlow et al., 2020). Its main aim is to en-hance students’ involvement in the learning process (Fredricks et al., 2004). It refers to the willingness and behaviors of individuals to actively mobilize cognitive resources for processing, integrating, and reflecting on information, which is crucial for understanding individual interactions with information, judgment formation, and decision-making (S. Li et al., 2020). The elaboration likelihood model (ELM) serves as a significant theoretical framework for comprehending cognitive engagement. This model differentiates between two paths of information processing: when individuals are highly motivated and capable, they engage in deep analysis through the “central path”, characterized by high cognitive engagement. In contrast, they may resort to the “peripheral path”, exhibiting low cognitive engagement by relying on the attractiveness of the information source or emotional cues for judgment (Petty & Cacioppo, 2012). Consequently, the degree of cognitive engagement influences how information is processed and affects the intensity and persistence of attitude change. Further research has demonstrated the extensive applicability of cognitive engagement across various fields, particularly in consumer behavior research, where it elucidates consumers’ processing of brand pages, product details, advertising content, and subsequent decision-making (Claffey & Brady, 2019; Ladeira et al., 2021; Ma et al., 2022; Zinkhan & Braunsberger, 2004). Diverse research perspectives have enriched the understanding of cognitive engagement. Some studies highlight its role as an individual’s ability to think critically and make decisions using effective strategies in complex informational contexts (S. Li et al., 2020), while others emphasize motivational factors, focusing on an individual’s interest and investment in specific information (Pohl, 2020). This aligns with the concept of “cognitive demand” proposed by Cacioppo et al., which underscores that an individual’s intrinsic tendency toward deep thinking is a key determinant of cognitive engagement (Cacioppo & Petty, 1982). In conclusion, within the specific context of KBSVs, this study conceptualizes cognitive engagement as the extent to which users focus on short video content and the level of cognitive processing they invest.

### 2.3. Expectations

Expectations are cognitive pre-assessments established before consumption decisions, influenced by various informational inputs such as past experiences, brand equity, and individual demand structures. These expectations shape perceptions of a product or service’s performance, quality, and overall experience (Oliver & Winer, 1987; Teas, 1993). In other words, expectation refers to an individual’s psychological assessment of the dif-ferent value dimensions of a product before they engage in consumption behavior (Teas, 1993). They serve as the cognitive framework for consumer decision-making, encompassing expectations of core product attributes, including technical parameters and quality standards (Antonides & Hovestadt, 2021; Curutchet et al., 2019; Nam et al., 2017), as well as expectations of additional values, such as service level and adaptability to specific usage scenarios. This process essentially represents a prejudiced psychological construction of the transaction value by consumers (Kahneman & Tversky, 2013). The formation of these expectations involves the information integration process of cognitive psychology, which includes the retrieval of experiences from memory, the influence of reference groups in social cognition, the encoding and decoding of marketing information, and the dynamic mapping of individual demand levels. Ultimately, this leads to the establishment of value judgment standards at the critical point of consumer decision-making (Krishnamurthy & Kumar, 2015; Schwarz, 2004; Sujan et al., 1986; Suomala, 2020). In summary, within the specific research context, this study conceptualizes expectations as users’ psychological anticipations regarding the quality and experience of knowledge products presented in KBSVs.

### 2.4. Knowledge Anxiety

The widespread adoption of the Internet has greatly improved access to knowledge; however, it has also led to the challenge of information overload (Hołyst et al., 2024). As individuals navigate this overwhelming influx of information, they are becoming more aware of their knowledge limitations and increasingly anxious about being left behind (R. Chen & Yan, 2023; Zhenlei et al., 2024a; Zhenlei et al., 2024b). In this rapidly evolving informational environment, knowledge anxiety has emerged as a common psychological response. This anxiety reflects the growing societal expectations for individuals’ knowledge and skills, alongside the difficulties of adapting to such swift changes (Hołyst et al., 2024). Knowledge anxiety is a complex concept, encompassing both a stable personality trait (trait anxiety) and a temporary, situation-dependent emotional state (situational anxiety) (Meijer, 2001). Trait knowledge anxiety indicates a general pre-disposition to feel anxious about one’s knowledge base. However, this study focuses specifically on situational knowledge anxiety, defined as the transient negative emotions—such as tension and unease—that arise when individuals perceive a gap between their existing knowledge and the knowledge necessary to keep up with social developments while engaging with KBSV (L. Wang, 2020; Zhenlei et al., 2024b). This state is often triggered by situational cues, such as exposure to new information or social comparisons with others (Hong et al., 2022; Naveed & Anwar, 2021). In this study, knowledge anxiety is framed not just as an emotional response but also as a motivational state that heightens users’ sensitivity to external cues, influencing their decision-making processes.

## 3. Research Model and Hypothesis Development

**Figure 2:**
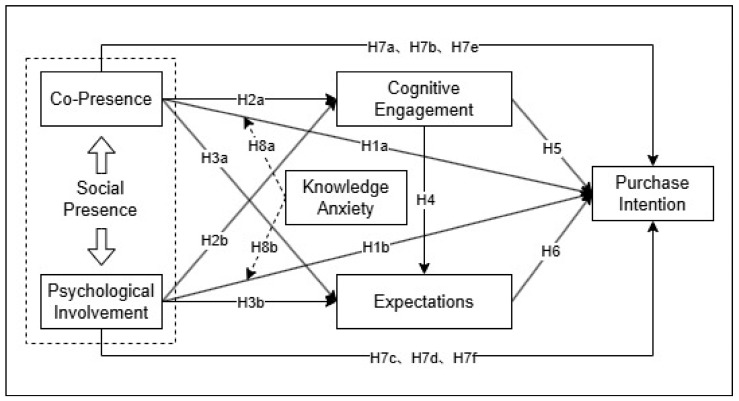
Conceptual model of the study.

Utilizing a moderated mediation model, as illustrated in Figure 2, this study examines the relationship between social presence and purchase intention within the context of KBSV. It reveals the indirect effects of cognitive engagement and expectations while concurrently evaluating the moderating role of knowledge anxiety in this intricate relationship.

### 3.1. Social Presence and Purchase Intention

Social presence serves as a crucial mechanism for enhancing non-contact social interaction (Cui et al., 2012). Short video platforms significantly improve users’ perception of the “presence” of creators and other viewers through video content, scrolling comments, engaging discussions in comment sections, and real-time purchase prompts, thereby fostering a sense of close virtual co-presence (W. Jiang & Chen, 2024). This social presence-driven non-contact interaction cultivates a strong group atmosphere and shared experience, effectively mitigating the loneliness and uncertainty users may encounter while making decisions independently (N. Li et al., 2024; Srivastava & Chandra, 2018). In such an interactive environment, users are more inclined to trust content creators and the products they endorse, thereby alleviating the psychological pressure associated with purchasing decisions and enhancing purchase intentions (Lu et al., 2015; R. Wang, 2024). Furthermore, research suggests that a heightened level of social presence enriches users’ social experiences and fosters a stronger sense of community belonging (Lee et al., 2023). This sense of belonging cultivates trust, making users more likely to support the communities with which they identify and the content creators within them through tangible actions (Hsu, 2017). Additionally, the interactive experience facilitated by social presence can enhance emotional resonance and gradually lead users to develop an emotional attachment to creators (Mühlhoff, 2014). This profound emotional connection increases users’ receptiveness to creators’ consumption recommendations and significantly lessens their concerns about potential risks when considering purchase decisions, ultimately driving purchase intention (Meng et al., 2021). In summary, this study predicts a substantial positive association between social presence in KBSVs and users’ purchase intentions for knowledge products.

**H1a.**  *Co-presence has a positive impact on purchase intention.*

**H1b.**  *Psychological involvement positively influences purchase intention.*

### 3.2. Social Presence and Cognitive Engagement

Social presence, which reflects a medium’s capacity to facilitate psychological engagement with others (J. Kim et al., 2016), plays a crucial role in capturing and maintaining users’ attention by enhancing interactivity and fostering a sense of presence (Y. L. Zhao et al., 2024). When users feel a sense of connection with creators in the short video environment, this social atmosphere enhances their perception of the content as more engaging and professional (Edwards et al., 2015). Users are motivated to focus on the ongoing content to avoid missing critical interactive cues, observe others’ responses, or anticipate feedback on their contributions. This drive for participation, stemming from co-presence, minimizes distractions and elevates both concentration levels and depth of thought (Zheng et al., 2024). Additionally, observing the views and behaviors of others may encourage users to reflect on and compare information (Kytle & Bandura, 1978). Related studies have corroborated these concepts. For instance, evidence from virtual reality learning research indicates that social presence positively influences cognitive engagement, largely due to the intrinsic alignment of measurement indicators (Dunmoye et al., 2024). Overall, this study posits that social presence within KBSVs will have a significant positive impact on users’ cognitive engagement.

**H2a.**  *Co-presence positively influences cognitive engagement.*

**H2b.**  *Psychological involvement positively influences cognitive engagement.*

### 3.3. Social Presence and Expectations

In the context of KBSV, social presence plays a pivotal role in shaping users’ expectations regarding products. First, when users perceive co-presence with video creators or fellow viewers, this sense of close social interaction enhances their trust and establishes a reference group (Weidlich et al., 2023). This reference group serves as a valuable guide for users’ social reference mechanisms, thereby elevating their expectations (Kühl et al., 2023; Ming et al., 2021). Additionally, when users encounter positive comments and high purchase numbers from the anchor or other users in the comment section, this interactivity fosters a perception of reliability in the product information and reduces perceived risk (Faradhilla et al., 2024), consequently leading to heightened expectations for quality assurance. Second, psychological involvement elevates expectations to a more emotional level. When users form positive emotional connections with video creators, they are inclined to project these emotions onto the recommended products (X. Wang et al., 2019), anticipating higher quality and value (Bonnefoy-Claudet & Ghantous, 2013). The combined effect of these two mechanisms—co-presence and psychological involvement—integrates users’ expectations of KBSV products with interactive experiences and emotional significance. In summary, this study posits that enhanced social presence in KBSVs will significantly elevate users’ expectations of knowledge product quality and experience.

**H3a.**  *Co-presence positively influences expectations.*

**H3b.**  *Psychological involvement positively influences expectations.*

### 3.4. Cognitive Engagement and Expectations

The degree of cognitive investment in KBSV content is a critical prerequisite for shaping users’ expectations of knowledge products. When users develop an interest in KBSV content and engage in deep cognitive processing, this heightened level of cognitive engagement reflects their intrinsic motivation to learn (Y. Shi et al., 2021), thereby fostering more positive expectations of knowledge products (Kuswanto et al., 2023). The process of cognitive engagement involves users actively constructing the value of the content (Messner, 2020), which plays a pivotal role in shaping their subsequent expectations. This aligns with Information Processing Theory, which posits that users’ cognitive engagement influences their attitudes and expectations (Salomon, 2012). Additionally, cognitive engagement may adjust users’ expectations through feedback loops; that is, after actively processing content, users may recalibrate their expectations for future content based on their current experiences (Oliver, 1980), such as anticipating knowledge payment products with greater depth and learning outcomes. This is linked to learning theory in consumer behavior (Schiffman et al., 2019), particularly cognitive learning theory, which asserts that users continuously learn and update their beliefs and expectations through experience (Hoch & Deighton, 1989). The distinctiveness of KBSV further emphasizes the significance of cognitive engagement in expectation formation. Unlike entertainment-oriented short videos, the core value of KBSV lies in effectively imparting knowledge and enhancing user understanding. Users’ ability to derive benefits from KBSV largely depends on the extent of their cognitive engagement. Only when users actively think, comprehend, and internalize knowledge points can they genuinely assess the value of the content and form realistic expectations of the knowledge products involved in KBSV. Therefore, this research posits that increased cognitive engagement within KBSV environments will have a significant positive impact on users’ expectation formation.

**H4.**  *Cognitive engagement positively affects expectations.*

### 3.5. Cognitive Engagement and Purchase Intention

The relationship between cognitive engagement and purchase intention is influenced by two fundamental mechanisms: enhanced information processing and the mitigation of cognitive dissonance. From the perspective of information processing theory, high cognitive engagement signifies that consumers allocate greater cognitive resources to information processing (Heddy et al., 2018). This in-depth processing enables consumers to gain a better understanding of product characteristics and benefits, allowing them to integrate this information into their existing knowledge framework and form more distinct product memories (Ross & Bettman, 1979). A comprehensive understanding of a product fosters the development of favorable consumer attitudes (Su et al., 2019), which subsequently increases the likelihood of purchase intention (Y. Li & Peng, 2021). According to cognitive dissonance theory, when consumers dedicate significant cognitive effort to comprehend a product or service, opting not to make a purchase may result in dissonance—a psychological discomfort stemming from the conflict between their cognitive investment and actual behavior (Hinojosa et al., 2016). To alleviate this discomfort and address sunk costs, consumers may be inclined to rationalize the purchase as worthwhile or to evaluate the product more positively, thereby enhancing their purchase intention and aligning their behavior with their prior cognitive investment (Kassarjian & Cohen, 1965; Kwak & Park, 2008). In summary, this study posits that higher levels of cognitive engagement in KBSV contexts will substantially increase users’ intention to purchase knowledge products.

**H5.**  *Cognitive engagement positively affects purchase intention.*

### 3.6. Expectations and Purchase Intention

Consumer behavior is significantly influenced by individuals’ assessments of potential benefits and costs, typically reflected in their perceived value of a product or service (Peng et al., 2019; Verhallen & Fred van Raaij, 1986). Within this framework, expectations play a crucial role. Expectations are subjective estimates of the utility that a product may provide before actual consumption (Teas, 1993). Higher user expectations often correlate with increased anticipated benefits and higher product ratings (Olshavsky & Miller, 1972; Suvittawat, 2024), thereby positively influencing the formation of purchase intentions (Mauri & Minazzi, 2013). Moreover, particularly in the e-commerce context, consumption decisions are frequently accompanied by various risks, including functional and financial risks (D. J. Kim et al., 2007). The perceived product value derived from user expectations can be viewed as the “benefit” aspect influencing behavioral decisions, while perceived purchase risk represents the “cost” aspect. Consequently, it can be inferred that when user expectations are elevated, the anticipated value benefits are more likely to surpass the perception of associated risks, positively impacting purchase intentions. Supporting this rationale, existing research indicates that expectations can elicit positive emotions in users, which subsequently affect their cognitive evaluations and behaviors (Hsieh & Yuan, 2019). In summary, this study posits that user expectations within KBSV environments will significantly enhance their willingness to purchase knowledge products.

**H6.**  *Expectations positively affect purchase intention.*

### 3.7. Mediating Role of Cognitive Engagement and Expectations

Based on the preceding theoretical discussion and hypothesis formulation, this study posits that the social presence perceived by users in KBSVs can effectively attract and sustain user attention, thereby positively influencing cognitive engagement (H2a, H2b). Furthermore, a heightened level of cognitive engagement not only reflects users’ intrinsic motivation but also enhances their value perception, creating a positive feedback loop that favorably impacts their expectations of knowledge products (H4). Ultimately, the establishment of user expectations will significantly elevate their purchase intention for knowledge products by improving their assessment of expected utility and eliciting positive emotional responses (H6). Consequently, cognitive engagement and expectations are proposed to serve as successive mediating variables through which social presence influences users’ intention to purchase knowledge products, thereby forming a serial mediation model.

**H7a.**  *Cognitive engagement mediates the effect of co-presence on purchase intention.*

**H7b.**  *Expectations mediate the effect of co-presence on purchase intention.*

**H7c.**  *Cognitive engagement mediates the effect of psychological involvement on purchase intention.*

**H7d.**  *Expectations mediate the effect of psychological involvement on purchase intention.*

**H7e.**  *Cognitive engagement and expectations play a serial mediating role in the effect of co-presence on purchase intention.*

**H7f.**  *Cognitive engagement and expectations play a serial mediating role in the effect of psychological involvement on purchase intention.*

### 3.8. Regulatory Role of Knowledge Anxiety

While knowledge anxiety is generally perceived as a negative emotional state, it can, under certain conditions, serve a positive moderating role in the relationship between social presence and purchase intention. This regulatory mechanism operates on multiple levels. First, from the perspective of compensatory motivation theory, individuals experiencing heightened knowledge anxiety are more likely to engage in proactive behaviors aimed at alleviating their discomfort, enhancing their competencies, or addressing perceived knowledge deficits (S. Kim & Chang, 2023; Koles et al., 2017). Consequently, interactions and recommendations from others are often perceived as effective solutions by those with high knowledge anxiety, thereby increasing their purchase intention. Second, the mechanism of social identity further supports this positive regulation. Individuals with elevated knowledge anxiety possess a stronger need for group belonging (Meuret et al., 2016). Thus, for these individuals, purchasing knowledge products that are recognized or utilized by their peer group becomes a means of integration, helping to mitigate feelings of isolation and uncertainty. Additionally, decision simplification mechanisms may also play a significant role. When faced with information overload and heightened knowledge anxiety, individuals frequently encounter decision-making difficulties and are more inclined to rely on external cues as heuristic shortcuts to alleviate cognitive burden and simplify their choices (Jia et al., 2020; Bennet & Bennet, 2008; Weirich, 2015). As a result, users experiencing high levels of knowledge anxiety tend to depend more heavily on the abundant social cues provided by social presence when making consumption decisions. This study posits that individuals with varying degrees of knowledge anxiety exhibit different compensation motives, group belonging needs, and decision-making challenges, leading to variations in their attention to social presence and purchasing behavior. When knowledge anxiety is high, users’ purchase intentions become increasingly reliant on interactions, group identity, and social cues offered by social presence. Conversely, when knowledge anxiety is low, the demand for interaction, group identity, and social cues diminishes, resulting in decreased attention to purchasing. In summary, the influence of social presence within KBSV on users’ intention to purchase knowledge products is contingent upon their level of knowledge anxiety, with a greater degree of knowledge anxiety strengthening this relationship.

**H8a.**  *Knowledge anxiety positively moderates the influence of co-presence on purchase intention.*

**H8b.**  *Knowledge anxiety positively moderates the influence of psychological involvement on purchase intention.*

## 4. Methodology

### 4.1. Data Acquisition

Data collection for this study specifically targeted individuals with prior experience viewing KBSV content. To ensure standardized and efficient sampling, the survey was conducted using Wenjuanxing (www.wjx.cn), a widely recognized online research platform in China. This platform facilitated both the distribution of the questionnaire and the collection of responses. In February 2025, the survey was deployed online, with invitations distributed to eligible participants drawn randomly from the platform’s user pool.

To improve response rates and ensure accurate data entry, a financial incentive of approximately 5 CNY was provided to participants who completed the questionnaire. To maintain data integrity, each IP address was restricted to a single submission. A total of 692 valid responses were initially collected. After excluding low-quality entries, such as those with uniform response patterns (for example, selecting the same option throughout) or unusually short completion times, 663 valid responses were retained for analysis. According to established methodological standards, the sample size should be at least ten times the number of questionnaire items (Kline, 2023). The final sample met this requirement, supporting the reliability of subsequent analyses.

### 4.2. Measures

All latent variables in this study were measured using established scales validated in prior research. Table 1 provides a comprehensive overview of the constructs, corresponding measurement items, and original source literature. To ensure the relevance of the items to the KBSV context, the original English scales were first translated into Chinese and then back-translated to verify accuracy. The wording was subsequently adapted to reflect the specific user experience on these platforms.

A pilot test with 30 participants was conducted to evaluate the clarity of the items and identify any potential issues. Based on the feedback received, minor modifications were made to enhance the comprehensibility of certain ambiguous items. The finalized questionnaire consisted of two sections: (1) demographic information, which included five items capturing respondents’ basic characteristics; and (2) measurements of latent variables, comprising 24 items that addressed co-presence, psychological involvement, cognitive engagement, expectations, knowledge anxiety, and purchase intention. All items were assessed using a 7-point Likert scale, ranging from 1 (“strongly disagree”) to 7 (“strongly agree”).

### 4.3. Data Analysis

Data analysis in this study was conducted using SPSS 25 and SmartPLS 4. SPSS 25 facilitated the generation of descriptive statistics and the execution of Harman’s single-factor test, while SmartPLS 4 served as the primary tool for partial least squares structural equation modeling to investigate the relationships and underlying mechanisms among the variables.

PLS-SEM is a variance-based structural equation modeling technique that is particularly effective for complex predictive models and theory development, especially when the goal is to explain the variance in the dependent variable (Hair et al., 2023). This method was selected for its robustness with non-normal data and its ability to handle both reflective and formative constructs, making it well-suited for testing the moderated mediation model in this study.

### 4.4. Respondent Demographic Characteristics

Table 2 presents the demographic profile of 663 respondents, offering a representative snapshot of the target user population. The sample exhibits a nearly even gender distribution, with females (52.2%) slightly outnumbering males (47.8%). The age distribution favors young adults, as the 26–35 age group represents the largest segment at 43.6%, followed by those aged 36–45 at 29.3%. Regarding educational attainment, most participants hold a bachelor’s degree (54.4%), indicating a well-educated sample. Monthly income primarily falls within the 3000–8000 CNY range (56.7%). Additionally, viewing frequency data shows that a significant portion of the sample consists of active users, with 60.3% watching KBSV at least once a day. The demographic profile of the respondents, which included a balanced gender distribution and a majority aged between 26 and 35 years, closely aligns with the user characteristics reported in the China Short Video Industry Insight Report 2020 (China Short Video Industry Insight Report, 2020). This alignment suggests that the sample is representative of the broader user population in China, providing a solid foundation for the external validity of model testing and hypothesis evaluation.

## 5. Results

### 5.1. Reliability and Validity Analysis

The reliability test is a fundamental step in the PLS-SEM analysis procedure, designed to assess the consistency and reliability of the measurement indicators for each latent variable. The measurement model exhibited strong reliability and validity, as detailed in Table 3, Table 4 and Table 5. Reliability was established through Cronbach’s α coefficients (0.832–0.899) and Composite Reliability (CR) values (0.900–0.925), all surpassing the acceptable threshold of 0.7 (Fornell & Larcker, 1981; Hair et al., 2023). Convergent validity was further substantiated, with factor loadings (0.822–0.899) and Average Variance Extracted (AVE) values (0.699–0.749), both exceeding the respective thresholds of 0.6 and 0.5 (Fornell & Larcker, 1981; Hair et al., 2011). Discriminant validity was assessed using two methods: the square root of the AVE for each variable was greater than its correlation with other variables, and all Heterotrait-Monotrait (HTMT) ratios remained below 0.85 (Henseler et al., 2014). These findings affirm the model’s robust reliability and validity.

### 5.2. Collinearity Analysis

In PLS-SEM analysis, the collinearity test ensures that predictor variables do not have high correlations, which helps prevent bias in estimating path coefficients. Standard guidelines indicate that if the VIF values of all internal models do not exceed 5, the risk of multicollinearity is considered low (Hair et al., 2011). As presented in Table 6, the VIF values in this study range from 1.089 to 2.146. Consequently, multicollinearity is unlikely to distort the results of the subsequent path analysis.

### 5.3. Path Analysis

**Figure 3:**
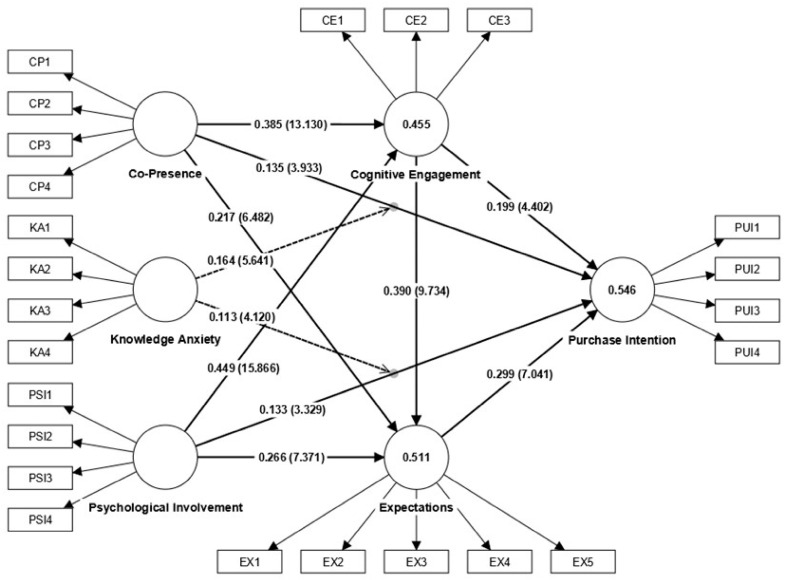
Analytical results of the model.

This study utilized bootstrapping procedures for path analysis. The statistical results are detailed in Table 7, and the model path relationships are depicted in Figure 3.

The direct effect analysis reveals that CP and PSI exert significant positive effects on PUI (H1a: β = 0.135, t = 3.933, *p* = 0.000; H1b: β = 0.133, t = 3.329, *p* = 0.001), CE (H2a: β = 0.385, t = 13.130, *p* = 0.000; H2b: β = 0.449, t = 15.866, *p* = 0.000), and EX (H3a: β = 0.217, t = 6.482, *p* = 0.000; H3b: β = 0.266, t = 7.371, *p* = 0.000), assuming that hypotheses H1a, H1b, H2a, H2b, H3a, and H3b are supported. Furthermore, CE has significant positive effects on EX (H4: β = 0.390, t = 9.734, *p* = 0.000) and PUI (H5: β = 0.199, t = 4.402, *p* = 0.000), assuming that hypotheses H4 and H5 are supported. EX also demonstrates significant positive effects on PUI (H6: β = 0.299, t = 7.041, *p* = 0.000), assuming that hypotheses H6 is supported.

The mediation effect analysis indicates that CE and EX serve as mediators in the pathways from CP to PUI (H7a: β = 0.077, t = 4.155, *p* = 0.000; H7b: β = 0.065, t = 4.743, *p* = 0.000) and from PSI to PUI (H7c: β = 0.090, t = 4.185, *p* = 0.000; H7d: β = 0.080, t = 4.929, *p* = 0.000), assuming that hypotheses H7a, H7b, H7c and H7d are supported. Additionally, CE and EX exhibit serial mediation roles in the pathways from CP to PUI (H7e: β = 0.045, t = 5.124, *p* = 0.000) and from PSI to PUI (H7f: β = 0.052, t = 5.243, *p* = 0.000), assuming that hypotheses H7e and H7f are supported.

The moderation effect analysis demonstrates that KA significantly moderates the strength of the relationships between CP and PUI (H8a: β = 0.164, t = 5.641, *p* = 0.000) as well as between PSI and PUI (H8b: β = 0.113, t = 4.120, *p* = 0.000), assuming that hypotheses H8a and H8b are supported.

In summary, this study finds that social presence positively influences users’ cognitive engagement, expectations, and purchase intention, based on the research questions. Cognitive engagement and expectations act as mediators—and sequential mediators—in the relationship between social presence and purchase intention. Additionally, knowledge anxiety positively moderates this pathway.

### 5.4. Model Explanatory and Prediction Ability

The quality of the structural model was evaluated using R^2^ and Q^2^ values, as presented in Table 8. R^2^ measures the explanatory power of the model by indicating the proportion of variance in the endogenous variable that is explained by its predictors. In contrast, Q^2^ evaluates the model’s predictive relevance. All R^2^ values exceeded the recommended threshold of 0.25 (Sarstedt et al., 2014), while Q^2^ values were above 0 (Shmueli et al., 2016), demonstrating robust explanatory and predictive validity of the model (Shmueli et al., 2019).

## 6. Discussion and Implications

### 6.1. Discussion

This study examines KBSV consumption behavior and user purchase intention formation by analyzing core driving mechanisms and individual moderating factors. Grounded in social presence theory, it explores the direct effects of co-presence and psychological involvement, along with their indirect effects mediated by cognitive engagement and expectations. Additionally, it investigates the moderating role of knowledge anxiety within the primary influence pathway. The following section provides a comprehensive analysis of the research findings.

The findings indicate that, within the KBSV context, users’ perceptions of social presence serve as a critical determinant influencing their intention to purchase knowledge products. This suggests that fostering a sense of co-presence through short videos can help alleviate users’ feelings of uncertainty and isolation during the decision-making process, thereby positively influencing their purchase intentions. Additionally, the psychological engagement that users develop through viewing and interaction acts as a powerful catalyst for purchasing tendencies by enhancing their emotional connection and sense of belonging to the creator or community. This conclusion aligns with previous research (N. Li et al., 2024; Lu et al., 2015), which posits that the social atmosphere and emotional bonds fostered by social presence are essential in stimulating users’ purchase intentions. How-ever, this study distinguishes itself by not treating social presence as a singular or vague concept, nor by merely categorizing it into dimensions based on different identity groups. Instead, it adopts a multidimensional theoretical perspective on social presence, allowing for a more nuanced distinction and empirical examination of its sources.

Social presence significantly enhances cognitive engagement. The attention and depth of thought that users invest in KBSV content are substantially influenced by the positive experiences associated with co-presence and psychological involvement. This key finding aligns with research in other media environments (Dunmoye et al., 2024), under-scoring the importance of social presence in engaging users’ cognitive resources and facilitating deeper information processing. The statistical analysis reveals that the emotional connection fostered by psychological involvement is crucial for encouraging users to engage in more profound cognitive processing. This contrasts with the findings of Ma et al., which suggested that positive emotions primarily drive behavioral and emotional engagement, while stimulating cognitive engagement is more challenging (Ma et al., 2022). However, evidence from cognitive neuroscience supports this study’s conclusions, indicating that psychological involvement is often linked to positive emotional responses, such as curiosity, pleasure, and resonance. Moderate positive emotions facilitate more flexible and creative information processing, leading users to deeper associations, integration, and reflection rather than mere superficial understanding (Fredrickson, 2004). This cognitive mechanism is not unique to knowledge consumption; it mirrors the evaluation process in other complex media domains, such as the arts, where viewers’ progression from simple identification to deeper understanding and reflection is essential for assessing the value of a creative work (Yuan & Sun, 2025). In contrast, attention generated solely by co-presence may lean more toward passive environmental monitoring. Consequently, psychological involvement proves to be more effective than basic co-presence in transforming users’ attention into high-quality cognitive engagement.

Social presence also positively influences user expectations. This indicates that users’ psychological assessments of product performance, quality, and experience, developed while viewing KBSVs, are directly affected by their level of co-presence and psychological engagement during media interactions. This finding aligns with previous studies (Kühl et al., 2023; Ming et al., 2021), which emphasize that social cues and emotional connections can effectively shape and enhance user expectations. This research extends beyond the examination of general social factors by employing social presence theory as a fundamental analytical framework, systematically integrating and deconstructing the intricate processes that shape these expectations. It offers a comprehensive theoretical perspective for understanding how user expectations are specifically shaped within social media contexts.

Both cognitive engagement and expectations positively impact users’ purchase intentions and mediate the relationship between social presence and purchase intentions. Specifically, social presence enhances users’ information processing and future value expectations by fostering an intimate and warm virtual environment, as well as facilitating positive interactions and feedback. This combination effectively strengthens users’ purchase intentions. This finding aligns with previous research (Hsieh & Yuan, 2019; Kwak & Park, 2008), which underscores the critical role of cognitive engagement and expectations in influencing consumer behavior. While the literature may not provide entirely consistent hypothetical models for direct comparison, this study significantly advances the understanding of the mediating roles of cognitive engagement and expectations, thereby enriching the relevant theoretical frameworks.

Cognitive engagement significantly shapes expectations, creating a mediation effect between social presence and purchase intention. This finding highlights the importance of social presence, cognitive engagement, and expectations in influencing purchase intention within a KBSV environment. Positive social experiences and the interactive atmosphere fostered by social presence enhance users’ cognitive engagement. When users focus more intently and engage in deeper thought, they actively construct and internalize the value of the content, generating feedback loops that transform high levels of cognitive engagement into positive expectations for future experiences. Ultimately, these enhanced, cognition-based expectations substantially increase users’ purchase intentions, completing the influence chain. This outcome provides a nuanced perspective that integrates cognitive processing and value evaluation, contributing to a better understanding of user purchasing behavior and facilitating targeted interventions within KBSV platforms.

Knowledge anxiety positively influences the relationship between social presence and purchase intention. Specifically, the impact of social presence on purchase intention is greater among individuals with higher levels of knowledge anxiety. Additionally, knowledge anxiety has a stronger moderating effect on the relationship between co-presence and purchase intention. This may be due to individuals with high knowledge anxiety often adhering more closely to social norms and experiencing increased decision-making pressure (Hong et al., 2022; Jia et al., 2020). The “visibility of others” provided by co-presence satisfies their need for social reference, validating their choices and facilitating group recognition (Baumeister & Leary, 2017; Meuret et al., 2016). Purchasing products endorsed by others has become an effective strategy to alleviate anxiety. This finding suggests that, in the context of KBSV, anxiety stemming from concerns about falling behind in knowledge is not solely negative; rather, it amplifies the influence of social cues on consumption decisions. This reflects the tendency of users with high knowledge anxiety to rely more heavily on external social cues to mitigate internal anxiety and guide their behavior. This research challenges the conventional view of knowledge anxiety as merely a learning motivation or negative emotion, providing new empirical evidence for applying social presence theory to understand the psychological states of specific users. It is crucial to consider the high correlation between cognitive engagement and expectations. Although our discriminant validity analyses confirmed that they are statistically distinct constructs, their strong positive relationship (r = 0.654) carries theoretical significance. This suggests that, in the realm of knowledge products, the process of becoming cognitively engaged—meaning deeply processing information—is closely tied to developing positive expectations about the product’s value. This finding supports our serial mediation model, where deep thinking naturally precedes and contributes to forming favorable future out-looks. However, this conceptual closeness calls for careful interpretation.

### 6.2. Implications

#### 6.2.1. Theoretical Implications

This study makes significant theoretical contributions.

First, it applies social presence theory to the rapidly evolving emerging media ecosystem of KBSV. Notably, the research does not treat social presence as a singular concept; instead, it adheres to its multidimensional composition theory to empirically test and confirm the independent effects and relative importance of the two core dimensions: co-presence and psychological involvement. By elucidating the roles of these dimensions in shaping users’ cognition, expectations, and behavioral intentions, this study provides empirical support for a more nuanced interpretation and assessment of social presence theory within specific media contexts, thereby enhancing the understanding of how social presence operates across different psychological levels.

Second, in contrast to earlier research that primarily focused on direct effects or single mediation paths, this study constructs and validates a serial mediation model incorporating cognitive engagement and user expectations. This model elucidates the intricate interactions through which social presence influences positive value expectations by promoting in-depth information processing among users, thereby indirectly enhancing their purchase intentions. This finding deepens the understanding of the formation of purchase intentions within consumer behavior theory and offers a more comprehensive and explanatory theoretical framework for understanding and predicting the purchase intentions of KBSV users.

Finally, this study innovatively integrates knowledge anxiety—an increasingly prevalent psychological state in the information age—into the social presence-purchase intention theoretical framework, confirming its positive regulatory role. Specifically, it finds that higher levels of knowledge anxiety enhance the influence of social presence cues on purchase intentions. This finding has two key implications. First, it elucidates the mechanism through which users’ internal psychological states amplify the impact of external cues during media interactions. Second, while previous research has primarily characterized knowledge anxiety as a negative emotional experience or a basic learning motivator, this study reveals its more complex and context-dependent function: in certain social information processing contexts, knowledge anxiety may not merely inhibit or motivate learning but can also serve as a catalyst that heightens individuals’ sensitivity to social presence cues, thereby reinforcing specific behavioral tendencies. This underscores the necessity for future research to reevaluate the multifaceted role of knowledge anxiety within human–computer interaction and social media contexts.

#### 6.2.2. Practical Implications

This study provides several practical implications for KBSV platform managers and content creators.

Firstly, it highlights important considerations for KBSV platform managers. Considering the substantial moderating role of knowledge anxiety, the platform can develop algorithms informed by user behavior, such as search patterns, content dwell time, interaction frequency, and responses to specific tags, to dynamically assess users’ levels of knowledge anxiety. Based on these assessments, the interface can adaptively modify the prominence of social cues. For instance, for users exhibiting high anxiety, the visibility and interactivity of real-time online user counts, recent purchase history, and highly rated comments can be enhanced. Conversely, for users displaying low anxiety, the emphasis may shift toward highlighting content depth, professional certifications, or personalized learning path recommendations.

Second, it offers practical implications for content creators. Given that cognitive engagement is essential for shaping expectations and driving purchases, content creators should prioritize the structuring and depth of their material. They should foster cognitive engagement among users through methods such as case analysis, interactive Q&A, and mind mapping. Additionally, creators are encouraged to incorporate thinking nodes or discussion guides within their videos to facilitate two-way communication, thereby enhancing the depth of users’ information processing.

Finally, it outlines practical implications that are relevant for both KBSV platform managers and content creators. Given the significance of psychological engagement, platforms can leverage AI technology to analyze the emotions expressed in user comments and provide creators with actionable feedback to optimize their content, thereby enhancing emotional connections with users. Concurrently, despite the advancements in AI, many short video creators have started utilizing AI-generated avatars and voiceovers. However, the uniformity of these virtual elements can diminish users’ sense of co-presence. To effectively bolster users’ co-presence, creators must present authentic and trustworthy personal images and genuinely share their experiences and values when recommending knowledge products.

## 7. Conclusions, Limitations, and Future Research

The objective of this study is to investigate the mechanisms of social presence, specifically co-presence and psychological involvement, on users’ purchase intentions for knowledge products within KBSV environments. Additionally, the study aims to develop a theoretical model that encompasses social presence, knowledge anxiety, cognitive engagement, expectations, and purchase intention. The findings indicate that social presence positively influences users’ cognitive engagement, expectations, and purchase intentions, with cognitive engagement and expectations serving as joint mediators of this relationship. Furthermore, knowledge anxiety was identified as a significant moderator of the effect of social presence on purchase intention. This integrated model enhances our understanding of the psychological and behavioral mechanisms operating in KBSV contexts and provides valuable insights for the development of more targeted marketing strategies.

This study has several limitations that should be acknowledged, indicating opportunities for future research.

First, the findings are based solely on a sample of Chinese users, which may limit their generalizability. The strong emphasis on interactive engagement in the collectivist context of Chinese platforms suggests that users’ perception of “social presence” may be closely linked to the relationship-building needs characteristic of high-context cultures. In contrast, users in Western, more individualistic cultures may prioritize autonomy and self-expression in their digital interactions. These cultural differences, along with platform-specific factors such as algorithmic recommendation logic and functional design, represent potential boundary conditions for the model. Future research should validate the model’s applicability and stability across diverse cultural and platform contexts.

Second, this study utilized a cross-sectional design, capturing cognitive engagement at a single point in time and not accounting for its dynamic evolution. Cognitive engagement is a process that likely develops through repeated interactions between users and creators, influencing subsequent expectations and purchase intentions. Future studies could benefit from adopting longitudinal or experimental methods to better elucidate causal pathways and potential feedback mechanisms.

Third, while the model includes several key psychological mechanisms, it may have overlooked other variables that could significantly impact purchase intention, such as users’ prior knowledge, perceived risk, or content genre preferences. Future research could expand the model to incorporate these factors, enhancing its explanatory power and robustness.

Fourth, this study conceptualized knowledge anxiety as a situational psychological state without distinguishing between its trait and state components. Future work could develop and validate measurement instruments that differentiate between “trait knowledge anxiety” and “situational knowledge anxiety” to explore their distinct moderating roles in user behavior.

Fifth, the data were collected from a single source, which raises the possibility of common method bias (CMB). Although procedural and statistical controls—including anonymous measurement, Harman’s single-factor test, and a full collinearity test—were implemented and revealed no significant issues, the potential for bias cannot be entirely ruled out. Future research could mitigate this risk by employing multi-source data or a multi-wave study design.

Finally, there is some conceptual overlap between cognitive engagement and expectations. Although the constructs passed discriminant validity tests, their high correlation suggests that future studies could explore alternative models or develop more precise measurement indicators to better distinguish between the in-depth processing of content and the resulting value-assessment mechanisms.

## Figures and Tables

**Table 1:** Measurement.

Construct	Items	Sources
Co-Presence	I felt as if the characters (anchors/other users) in the KBSVs were right next to me.	(Gefen & Straub, 2004; Zheng et al., 2024)
	I feel as if the characters (anchors/other users) in the KBSVs are communicating with me in the same place.	
	I feel a connection between the characters (anchors/other users) in KBSVs and me across time and space.	
	I feel as if I am in the world created by the characters (anchors/other users) in the KBSVs.	
Psychological Involvement	I can feel social in KBSV.	(Animesh et al., 2011; J. Kim et al., 2016; Sun et al., 2019)
	I can feel warmth in KBSV.	
	I can feel enthusiasm in KBSV.	
	I can feel intimacy in KBSV.	
Cognitive Engagement	KBSVs inspired me to delve further into the knowledge.	(Ma et al., 2022; Barlow et al., 2020)
	KBSVs make me think about the principles and applications behind the knowledge.	
	KBSVs make me think frequently about the various points of knowledge related to them.	
Expectations	The content presentation of this knowledge product will be very exciting.	(Nam et al., 2017)
	The user experience of this knowledge product will be very good.	
	The information provided by this knowledge product will be of practical help to me.	
	The content presentation of this knowledge product will comply with the knowledge dissemination standards.	
	The information of this knowledge product will have lasting reference value.	
Knowledge Anxiety	Knowledge is updated too quickly, disrupting my normal study life.	(R. Chen & Yan, 2023; Naveed & Anwar, 2021; Zhenlei et al., 2024b)
	I often feel that my knowledge structure is not comprehensive enough and there is a gap between me and others.	
	I feel uncertain when judging the credibility of knowledge content.	
	I worry about missing something important due to information overload.	
Purchase Intention	I would consider purchasing these knowledge products.	(Peng et al., 2019; R. Wang, 2024)
	I am very likely to purchase these knowledge products.	
	I will purchase these knowledge products soon.	
	If I had enough time, energy, and money, I would be willing to purchase these knowledge products.	

**Table 2:** Respondent demographic characteristics (N = 663).

	Items	Frequency	Proportion
Gender	Male	317	47.8%
	Female	346	52.2%
Age (in years)	18–25	142	21.4%
	26–35	289	43.6%
	36–45	194	29.3%
	>45	38	5.7%
Education	High school or below	74	11.2%
	Three-year college	172	25.9%
	Undergraduate	361	54.4%
	Postgraduate or above	56	8.4%
Monthly income (CNY/Yuan)	<3000	35	5.3%
	3000–8000	376	56.7%
	8000–13,000	184	27.8%
	>13,000	68	10.3%
Frequency of watching KBSV	Multiple times per day	163	24.6%
	Once a day	237	35.7%
	Multiple times per week	185	27.9%
	Once a week	78	11.8%

**Table 3:** Reliability and validity analysis.

Constructs	Item	Factor Loadings	Cronbach’s Alpha	CR	AVE
Co-Presence (CP)	CP1	0.899	0.885	0.921	0.744
	CP2	0.860			
	CP3	0.846			
	CP4	0.842			
Psychological Involvement (PSI)	PSI1	0.833	0.856	0.903	0.699
	PSI2	0.822			
	PSI3	0.835			
	PSI4	0.853			
Cognitive Engagement (CE)	CE1	0.870	0.832	0.900	0.749
	CE2	0.866			
	CE3	0.861			
Expectations (EX)	EX1	0.839	0.899	0.925	0.713
	EX2	0.848			
	EX3	0.851			
	EX4	0.860			
	EX5	0.823			
Knowledge Anxiety (KA)	KA1	0.837	0.866	0.909	0.713
	KA2	0.854			
	KA3	0.844			
	KA4	0.843			
Purchase Intention (PUI)	PUI1	0.827	0.861	0.906	0.706
	PUI2	0.857			
	PUI3	0.827			
	PUI4	0.850			

**Table 4:** Discriminant validity (Fornell Larcker Criterion).

	CP	PSI	CE	EX	KA	PUI
CP	0.862					
PSI	0.306	0.836				
CE	0.522	0.567	0.865			
EX	0.502	0.553	0.654	0.844		
KA	0.244	0.146	0.172	0.177	0.844	
PUI	0.475	0.502	0.587	0.624	0.299	0.840

**Table 5:** Discriminant Validity (HTMT Criterion).

	CP	PSI	CE	EX	KA	PUI
CP						
PSI	0.350					
CE	0.608	0.670				
EX	0.562	0.631	0.755			
KA	0.278	0.170	0.203	0.202		
PUI	0.544	0.583	0.693	0.709	0.345	

**Table 6:** VIF Value of the Inner Model Matrix.

	CP	PSI	CE	EX	KA	PUI
CP			1.103	1.375		1.579
PSI			1.103	1.473		1.648
CE				1.835		2.146
EX						2.055
KA						1.089
PUI						
KA × CP						1.136
KA × PSI						1.152

**Table 7:** Hypothesis testing.

Paths	Hypotheses	Path Coefficients β-Values	*t*-Values	*p*-Values	Confidence Interval		Decision
					2.5%	97.5%	
Direct effects							
CP → PUI	H1a	0.135	3.933	0.000	0.066	0.201	Supported
PSI → PUI	H1b	0.133	3.329	0.001	0.055	0.212	Supported
CP → CE	H2a	0.385	13.130	0.000	0.326	0.441	Supported
PSI → CE	H2b	0.449	15.866	0.000	0.392	0.503	Supported
CP → EX	H3a	0.217	6.482	0.000	0.153	0.283	Supported
PSI → EX	H3b	0.266	7.371	0.000	0.194	0.335	Supported
CE → EX	H4	0.390	9.734	0.000	0.311	0.468	Supported
CE → PUI	H5	0.199	4.402	0.000	0.111	0.290	Supported
EX → PUI	H6	0.299	7.041	0.000	0.218	0.383	Supported
Mediation effects							
CP → CE → PUI	H7a	0.077	4.155	0.000	0.042	0.115	Supported
CP → EX → PUI	H7b	0.065	4.743	0.000	0.040	0.094	Supported
PSI → CE → PUI	H7c	0.090	4.185	0.000	0.049	0.133	Supported
PSI → EX → PUI	H7d	0.080	4.929	0.000	0.050	0.114	Supported
CP → CE → EX → PUI	H7e	0.045	5.124	0.000	0.029	0.063	Supported
PSI → CE → EX → PUI	H7f	0.052	5.243	0.000	0.035	0.073	Supported
Moderating effects							
KA × CP → PUI	H8a	0.164	5.641	0.000	0.108	0.221	Supported
KA × PSI → PUI	H8b	0.113	4.120	0.000	0.058	0.165	Supported

**Table 8:** R^2^ value and Q^2^ value.

	R^2^	Q^2^ Predict
Cognitive Engagement (CE)	0.455	0.451
Expectations (EX)	0.511	0.424
Purchase Intention (PUI)	0.546	0.437

## Data Availability

The original contributions presented in the study are included in the article. Further inquiries can be directed to the corresponding author.
